# Early mortality experience in a large military cohort and a comparison of mortality data sources

**DOI:** 10.1186/1478-7954-8-15

**Published:** 2010-05-24

**Authors:** Tomoko I Hooper, Gary D Gackstetter, Cynthia A LeardMann, Edward J Boyko, Lisa A Pearse, Besa Smith, Paul J Amoroso, Tyler C Smith

**Affiliations:** 1 Department of Preventive Medicine and Biometrics, Uniformed Services University of the Health Sciences, Bethesda, Maryland, USA; 2 Analytic Services Inc., Arlington, Virginia, USA; 3 Department of Defense Center for Deployment Health Research, Naval Health Research Center, San Diego, California, USA; 4 Seattle Epidemiologic Research and Information Center, Department of Veterans Affairs Puget Sound Health Care System, Seattle, Washington, USA; 5 Mortality Surveillance Division, Armed Forces Medical Examiner System, Rockville, Maryland, USA; 6 Madigan Army Medical Center, Fort Lewis, Washington, USA

## Abstract

**Background:**

Complete and accurate ascertainment of mortality is critically important in any longitudinal study. Tracking of mortality is particularly essential among US military members because of unique occupational exposures (e.g., worldwide deployments as well as combat experiences). Our study objectives were to describe the early mortality experience of Panel 1 of the Millennium Cohort, consisting of participants in a 21-year prospective study of US military service members, and to assess data sources used to ascertain mortality.

**Methods:**

A population-based random sample (n = 256,400) of all US military service members on service rosters as of October 1, 2000, was selected for study recruitment. Among this original sample, 214,388 had valid mailing addresses, were not in the pilot study, and comprised the group referred to in this study as the invited sample. Panel 1 participants were enrolled from 2001 to 2003, represented all armed service branches, and included active-duty, Reserve, and National Guard members. Crude death rates, as well as age- and sex-adjusted overall and age-adjusted, category-specific death rates were calculated and compared for participants (n = 77,047) and non-participants (n = 137,341) based on data from the Social Security Administration Death Master File, Department of Veterans Affairs (VA) files, and the Department of Defense Medical Mortality Registry, 2001-2006. Numbers of deaths identified by these three data sources, as well as the National Death Index, were compared for 2001-2004.

**Results:**

There were 341 deaths among the participants for a crude death rate of 80.7 per 100,000 person-years (95% confidence interval [CI]: 72.2,89.3) compared to 820 deaths and a crude death rate of 113.2 per 100,000 person-years (95% CI: 105.4, 120.9) for non-participants. Age-adjusted, category-specific death rates highlighted consistently higher rates among study non-participants. Although there were advantages and disadvantages for each data source, the VA mortality files identified the largest number of deaths (97%).

**Conclusions:**

The difference in crude and adjusted death rates between Panel 1 participants and non-participants may reflect healthier segments of the military having the opportunity and choosing to participate. In our study population, mortality information was best captured using multiple data sources.

## Background

Complete and accurate ascertainment of mortality is critically important in any longitudinal study. Death is an objective outcome measure that is captured in multiple medical and administrative data sources and provides an assessment of the overall health of a population, as well as patterns and trends related to specific causes of excess or reduced mortality. This paper describes the early mortality experience of the Millennium Cohort Study, a large prospective cohort study of individuals who are serving or have served in the US military [1], and compares data sources used for mortality ascertainment.

The Millennium Cohort Study was launched in 2001 in response to a recommendation by the Institute of Medicine that a prospective cohort study be undertaken to better understand any health effects related to military service [1,2]. Following three enrollment phases, 150,597 individuals consented and enrolled as Cohort members and participated in at least one Web-based or postal survey of physical and mental health status, health risk behaviors, and military deployment experience. This is the first large, population-based study to collect longitudinal data on military personnel from all service branches and components. Self-reported survey data is collected every three years (continuing through 2022), even after separation from active service, to evaluate possible health outcomes related to deployment, military occupations and exposures, and military service in general. Detailed descriptions of Millennium Cohort Study methodology have been published elsewhere [1,3]. Previous investigations have found no determinants for enrollment bias based on differences in health care utilization [4]; established the reliability of self-reported occupation [5] and self-reported vaccinations [6,7]; and indicated reliable reporting with respect to test/retest with high internal consistency for the standardized instruments included within the questionnaires [8].

The four data sources used to ascertain mortality within the Millennium Cohort were: 1) the National Center for Health Statistics National Death Index (NDI), 2) the Social Security Administration Death Master File (SSA-DMF), 3) the Department of Veterans Affairs (VA) data files, and 4) the Department of Defense Medical Mortality Registry (DoD-MMR). We describe the early mortality experience of the first enrollment panel (Panel 1) of the Cohort from July 2001 to December 2006 using three of these data sources (NDI was excluded because of the long lag time in posting deaths) and compare crude and adjusted death rates as well as frequency distributions between the participating and the non-participating members of the invited sample to assess any differences. In order to examine the utility of all four data sources for ongoing mortality ascertainment in our study population, we compared the number of deaths identified by each source from 2001 to 2004.

With the United States currently engaged in two major conflicts, there is great public interest in the health effects of military service, and in particular, any latent adverse effects of combat on the men and women who serve their country. Early tracking of mortality is important in this occupational group because of unique exposures (e.g., deployment to many regions of the world, as well as combat experiences) to establish a baseline for all-cause and cause-specific mortality over time. Additionally, because the Cohort includes a substantial proportion of Reserve and National Guard members and increasing numbers of individuals who separate from active military service and re-enter civilian life, this study population encompasses more than the narrowly defined active-duty military community. The study participants represent diverse socioeconomic strata and race/ethnicities and come from many different geographic regions of the country. Oversampling of women and Reserve/Guard members by design gives us insight into groups of particular interest to military leaders and the general public. Finally, describing the relative utility of available data sources for mortality ascertainment in our study population would benefit other researchers leveraging the rich array of electronic data from among the largest health maintenance organizations (DoD and VA) in the country.

## Methods

### Study population

The study population, Panel 1 of the Millennium Cohort, consists of 77,047 consenting participants enrolled between July 1, 2001, and June 30, 2003. An overview of the baseline Panel 1 Cohort was published in 2007 [3]. The population-based random sample of US service members selected to participate in the study (n = 256,400) represented approximately 11.3% of 2.2 million service members on service rosters as of October 1, 2000. Members of the Reserve Force and National Guard (Reserve/Guard), women, and those deployed to Southwest Asia, Bosnia, or Kosovo between 1998 and 2000 were oversampled to ensure adequate statistical power to assess rare outcomes in these smaller subgroups. Of the 256,400 in the original sample, 214,388 had valid mailing addresses and were not in the pilot study and comprised the group we refer to as the invited sample (n = 214,388). The participation rate for the baseline Cohort was 36% for those eligible and able to be contacted in the aftermath of challenges arising from the September 11, 2001, terrorist attack and anthrax mail threat [3]. Non-participants had the opportunity to enroll in the study, but either declined (n = 4,796), chose not to respond (n = 129,887), or were ineligible for other reasons (n = 2,658). Panel 1 participants completing the baseline survey were previously reported to be generally similar to the US military as a whole, but slightly more likely to be older, married, more educated, and officers [3].

### Data sources

#### National Death Index

The NDI is a central repository of automated US death records maintained by the National Center for Health Statistics (NCHS) and has been described as a comprehensive source of accurate and complete mortality data for research purposes [9-18]. It contains death certificate information reported by each state's vital statistics office since 1979, as well as capturing deaths occurring in the District of Columbia, Puerto Rico, and the Virgin Islands [18]. Sensitivity (correctly identifying deaths) has been reported to range between 87.0% and 97.9% [17] and is dependent on the type and quality of identifying information available for use in matching algorithms, with the Social Security number as the most important factor [10-13,17]. Some variability in sensitivity has been reported by race and gender, with men more likely to be identified as deceased than women, and Caucasians more likely to be identified than those belonging to other racial or ethnic groups [10,11,13,17]. Although NDI has been cited as the "gold standard" [17,19] for death ascertainment, it is not without its limitations, the most important of which is the considerable lag time (12 to 24 months) in posting deaths to this registry [19]. In addition, deaths occurring outside the United States and its territories are not captured, and fees related to the application process and data searches are a disadvantage, particularly for large studies [18,19]. However, one distinct advantage of this data source is the option to obtain cause-of-death information using NDI Plus [17].

To ascertain deaths among Panel 1 Cohort members from January 2001 through December 2004, the following information was sent to NCHS: Social Security number; first, middle, and last name; gender; and date of birth. Data received from NCHS in November 2006 included identifiers listed above, as well as state of death, death certificate number, and other matching variables.

#### Social Security Administration Death Master File

The SSA-DMF is another widely used source of vital status information on individuals enrolled in the US Social Security program and includes deaths since 1936 based on notification by family members, funeral directors, financial institutions, US Postal Service, and other federal and state agencies [17,19-21]. This database was established in 1988 as a public use resource with mortality information extracted from the SSA master database of all enrolled individuals (beneficiaries and non-beneficiaries) [9,10,20]. Monthly updates to SSA-DMF are made based on information contained within other SSA databases [19,21]. Sensitivity has been reported to range between 83% and 95% [19], with more complete identification of deaths among older individuals (93-96% of those aged 65 years and older) [20]. Completeness of mortality information depends on reporting to the SSA, with potentially less incentive for individuals ineligible to receive benefits (requires 10 years of work in the United States), such as younger and foreign-born decedents [21]. Compared with NDI, advantages include free Internet-based search capability, frequent updates to the database, and the inclusion of deaths occurring outside the United States and its territories.

For the Millennium Cohort Study, the SSA-DMF is accessed through the Defense Manpower Data Center (DMDC), Seaside, Calif., under an interagency agreement. Beginning October 1, 2000, DMDC has provided SSA-DMF mortality data (first, middle, and last name; Social Security number; date of birth; and date of death) on a monthly basis for all service members invited to participate in the Millennium Cohort Study whose Social Security number matched a record in the SSA-DMF. SSA-DMF data used for this study were acquired in January 2007 and included deaths through December 31, 2006.

#### Department of Veterans Affairs mortality datasets

The VA is another important national resource for mortality information since military veterans comprise a large proportion of the US population (approximately 12.7% aged 18 years and older based on the 2000 US census) [22]. The VA uses multiple data sources, in-house and from other agencies, to ascertain mortality in its veteran population [15,17,19,23-25]. The Beneficiary Identification and Records Locator Subsystem (BIRLS) is a key VA data source containing records of all beneficiaries, including veterans whose family members have applied for death benefits. The BIRLS Death File is a subset of this database and, despite primary reliance on survivor reporting, sensitivity has been reported to range from 80% to 96.5% [11,15,23] in veteran populations. The BIRLS Death File is supplemented by the Medical SAS Inpatient Datasets (MSID) [19], formerly the Patient Treatment Files, which include deaths occurring in or shortly after discharge from Veterans Health Administration hospitals.

The VA also routinely obtains mortality information from SSA-DMF and the Medicare Vital Status File for Medicare-enrolled veterans (since 1999). Accuracy and completeness in mortality ascertainment based on multiple databases have proved comparable to NDI [19]. For this study, mortality ascertainment at the VA was based on BIRLS, MSID, and SSA-DMF.

VA mortality data for the invited sample, matched using Social Security numbers, were received for this study in January 2007 and consisted of all deaths that occurred prior to the end of December 2006. The VA provided date of birth, date of death, and Social Security number for each decedent.

#### Department of Defense Medical Mortality Registry

The DoD-MMR is maintained in the Mortality Surveillance Division of the Armed Forces Medical Examiner System (AFMES), currently part of the Armed Forces Institute of Pathology (AFIP). The DoD-MMR contains detailed information on all US military active-duty deaths, including Reserve and National Guard members in an activated status, regardless of where the death occurred [26]. Data collection began in 1995, with near complete capture since 1998. There are two main strengths of this source: It is extremely current, with a direct connection to military casualty systems; and, unlike NDI and SSA-DMF, which are limited to death certificate information, it is a rich source of data, including information from autopsy reports, medical records, and police or investigative reports. The main limitation of the DoD-MMR is that deaths among veterans who have been separated for more than 120 days, reservists between activations, and military retirees are not captured. Because many veterans have also served as contractors in the current conflicts, records of autopsies performed by the AFMES on civilians and contractors killed overseas were also reviewed, and data were included when a match was confirmed.

Data from DoD-MMR, matched using Social Security numbers, were received for this study in April 2007 and included deaths that occurred among invited participants prior to April 15, 2007. AFIP provided date of birth; date of death; Social Security number; first, middle, and last name; cause of death; and military-specific variables.

#### Millennium Cohort mortality ascertainment

Unique identifiers for the entire baseline invited sample were submitted to three data sources (DMDC/SSA-DMF, VA, and AFIP/DoD-MMR) to ascertain deaths through December 31, 2006. Unique identifiers were also submitted to NCHS/NDI for enrolled Cohort members only, and NCHS provided information on deaths through December 31, 2004. In addition to describing the early mortality experience of enrolled Cohort members, the purpose of obtaining death information was threefold: 1) to remove decedents from the ongoing contact list of Cohort members; 2) to assess representativeness of the Cohort compared to the invited sample; and 3) to compare the utility of these specific mortality data sources.

For purposes of this study, a positive match between Cohort data and information provided by each mortality data source was defined as exact agreement on two of three personal identifiers consisting of Social Security number, first and last name, and full date of birth. NDI was not used as a gold standard because it only captures deaths that occur within the US and its territories, and our study population consisted of many service members stationed overseas.

### Analysis

Using three of the data sources, excluding NDI, crude death rates and 95% confidence intervals were calculated for Panel 1 participants and non-participants, as well as the invited sample (participants plus non-participants) from July 1, 2001, through December 31, 2006. Using person-year denominators, individuals were censored at time of death or the end of the observation period, and confidence intervals for rate estimates were based on a Poisson distribution. In order to more fully delineate any differences in the death rates for Panel 1 participants and non-participants, we compared age- and sex-adjusted (direct standardization) overall death rates and also examined the distribution of mortality by demographic and military service variables, categorized as follows: gender (male, female); age (based on birth year: pre-1960, 1960-1969, 1970-1979, 1980 or later); race/ethnicity (white non-Hispanic, black non-Hispanic, other); marital status (not married, married, divorced); education (high school or less, some college, bachelor's degree, advanced degree); service component (Reserve/National Guard, active duty); branch of service (Army, Air Force, Navy/Coast Guard, Marine Corps); military pay grade (enlisted, officer); occupational category (combat specialists, health care specialists, service supply and functional support, other); deployment experience (pre-2001, 2001-2006, pre-2001 and 2001-2006, none); and separation from military service (no, yes). Finally, we calculated age-adjusted, category-specific mortality rates per 100,000 person-years using direct standardization for Panel 1 participants and non-participants. We did not adjust for sex because of small numbers (<5) in several strata.

To assess the utility of the four sources of mortality data, deaths ascertained among Panel 1 participants were compared by source. Because of the lag time associated with NDI, deaths identified only through December 31, 2004, could be compared. We again examined the distribution of mortality by demographic and military service characteristics and graphically displayed counts and overlap by data source using a Venn diagram.

This study was reviewed and approved by institutional review boards at the participating organizations and conducted in accordance with federal and institutional regulations pertaining to research involving human participants (Protocol NHRC.2000.007).

## Results

Among the 214,388 individuals in the invited sample, 209,146 (97.6%) were presumed alive as of June 30, 2001, and had complete demographic data. Of these 209,146 invited service members, 76,960 enrolled in the study between July 1, 2001, and June 30, 2003, and the remaining 132,186 were non-participants.

Using data from three sources (SSA-DMF, VA mortality data sets, and DoD-MMR), there were 341 deaths identified among Panel 1 participants between July 2001 and December 2006, resulting in a crude death rate of 80.7 per 100,000 person-years (95% CI: 72.2,89.3). By comparison, 820 deaths were identified among Panel 1 non-participants for a crude death rate of 113.2 per 100,000 person-years (95% CI: 105.4, 120.9) over the same time period. The crude death rate for the invited sample was 101.2 per 100,000 person-years (95% CI: 95.4, 107.0). Applying direct standardization methods, the age- and sex-adjusted overall death rate for Panel 1 participants was 78.0 deaths per 100,000 person-years (95% CI: 68.3, 87.7) compared to 121.4 deaths per 100,000 person-years (95% CI: 112.9, 129.8) for Panel 1 non-participants.

The distribution of mortality through December 2006 among Panel 1 participants and non-participants is presented in Table 1. Men, older individuals (birth year prior to 1960), non-Hispanic whites, divorced, high school education or less, Reserve/National Guard members, Army, combat specialists, those not deployed or deployed prior to 2001, and those not separated from military service were overrepresented among the decedents of both Panel 1 participants and non-participants.

**Table 1:** Distribution of Mortality by Demographic and Military Characteristics Comparing Panel 1 Participants and Non-Participants for the Millennium Cohort Study, United States, July 1, 2001-December 31, 2006

Baseline Characteristics	Panel 1 Participants^a^		Panel 1 Non-Participants^b^	
	
	Total Sample *n *= 76,960 %^d^	Deceased^c^ *n *= 341 %^d^	Total Sample *n *= 132,186 %^d^	Deceased^c^ *n *= 820 %^d^
Gender				
Male	73.2	80.1	78.0	88.5
Female	26.8	19.9	22.0	11.5
Birth year				
Pre-1960	21.6	43.1	13.2	27.3
1960-1969	37.9	30.8	29.3	22.9
1970-1979	34.6	21.1	46.7	39.3
1980 or later	5.9	5.0	10.9	10.5
Race/ethnicity				
White non-Hispanic	69.6	74.8	63.9	65.0
Black non-Hispanic	13.8	11.4	20.9	20.2
Other	16.6	13.8	15.2	14.8
Marital status				
Not married	30.1	26.1	43.1	42.2
Married	63.1	65.4	51.3	49.6
Divorced	6.9	8.5	5.7	8.2
Education				
High school or less	48.9	49.3	61.0	66.0
Some college	25.5	25.8	24.6	19.9
Bachelor's degree	16.5	17.3	10.1	10.1
Advanced degree	9.1	7.6	4.3	4.0
Service component				
Reserve/National Guard	43.1	57.2	43.4	47.3
Active duty	56.9	42.8	56.6	52.7
Branch of service				
Army	47.4	50.7	43.5	51.8
Air Force	29.1	29.9	29.8	22.4
Navy/Coast Guard	18.5	14.7	19.9	18.4
Marine Corps	5.1	4.7	6.8	7.3
Military pay grade				
Enlisted	77.0	76.8	87.8	88.7
Officer	23.0	23.2	12.2	11.3
Occupational category				
Combat specialists	20.0	26.1	21.3	27.7
Health care specialists	10.4	9.1	7.6	6.3
Service supply and functional support	28.7	27.6	26.6	23.4
Other	40.9	37.2	44.5	42.6
Deployment experience^e^				
Pre-2001	18.9	22.3	18.3	23.2
2001-2006	21.7	16.4	21.5	13.5
Pre-2001 and 2001-2006	18.6	11.4	16.3	11.5
None	40.8	49.9	43.9	51.8
Separated from military service^f^				
No	76.5	84.5	73.8	83.2
Yes	23.5	15.5	26.2	16.8

^a ^Includes Panel 1 Millennium Cohort participants who enrolled in the study between July 1, 2001, and June 30, 2003, and had complete demographic data.

^b ^Includes service members who had complete demographic data, were presumed alive as of June 30, 2001, were able to be contacted by mail and were invited to enroll in the first panel of the Millennium Cohort Study, but either declined, did not respond, or were ineligible.

^c ^Data sources include Armed Forces Institute of Pathology, Department of Veterans Affairs, and Defense Manpower Data Center.

^d ^Percents may not sum to 100 due to rounding.

^e ^Deployment category "Pre-2001" refers to individuals deployed to the 1991 Gulf War or to Bosnia, Kosovo, or Southwest Asia between January 1, 1998, and September 30, 2000; the category "2001-2006" includes individuals deployed in support of the military operations in Iraq and Afghanistan between September 1, 2001, and December 31, 2006.

^ f ^Separation (retirement or early separation) from military service as of December 31, 2006 (excludes death as a reason for separation).

Age-adjusted, category-specific rates are presented in Table 2 to compare differences between Panel 1 participants and non-participants. These rates are standardized to the total military population in October 2000 and cannot be used other than to directly compare the two population subgroups. In all categories, the rates are consistently higher among study non-participants.

**Table 2:** Age-Adjusted, Category-Specific Death Rates^a ^Comparing Panel 1 Participants and Non-Participants for the Millennium Cohort Study, United States, July 1, 2001-December 31, 2006

Baseline Characteristics	Panel 1 Participants^b^ *n *= 76,960 No. of deaths (per 100,000 person-years)	Panel 1 Non-Participants^c^ *n *= 132,186 No. of deaths (per 100,000 person-years)
Gender		
Male	82.2	132.1
Female	56.4	62.8
Race/ethnicity		
White non-Hispanic	77.2	117.6
Black non-Hispanic	61.7	115.4
Other	66.8	111.2
Marital status		
Not married	84.2	125.0
Married	75.9	97.4
Divorced	57.0	110.0
Education		
High school or less	85.3	135.5
Some college	66.6	87.9
Bachelor's degree	65.2	92.1
Advanced degree	35.2	56.2
Service component		
Reserve/National Guard	80.3	112.1
Active duty	63.6	111.4
Branch of service		
Army	78.7	137.8
Air Force	64.9	81.5
Navy/Coast Guard	60.4	110.2
Marine Corps	73.2	167.2
Military pay grade		
Enlisted	77.4	121.4
Officer	64.0	81.5
Occupational category		
Combat specialists	104.5	150.7
Health care specialists	60.0	88.7
Service supply and functional support	65.8	98.3
Other	69.7	115.7
Deployment experience^d^		
Pre-2001	73.7	147.4
2001-2006	61.7	71.5
Pre-2001 and 2001-2006	46.3	92.7
None	77.8	130.9
Separated from military service^e^		
No	80.2	129.3
Yes	51.8	77.2

^a ^Number of deaths per 100,000 person years between Jul 1, 2001 and Dec 31, 2006, adjusted for birth year, standardized to the total US military as of October 1, 2000. Data sources include Armed Forces Institute of Pathology, Department of Veterans Affairs, and Defense Manpower Data Center.

^b ^Includes Panel 1 Millennium Cohort participants who enrolled in the study between July 1, 2001, and June 30, 2003, and had complete demographic data.

^c ^Includes service members who had complete demographic data, were presumed alive as of June 30, 2001, were able to be contacted by mail and were invited to enroll in the first panel of the Millennium Cohort Study, but either declined, did not respond, or were ineligible.

^d ^Deployment category "Pre-2001" refers to individuals deployed to the 1991 Gulf War or to Bosnia, Kosovo, or Southwest Asia between January 1, 1998, and September 30, 2000; the category "2001-2006" includes individuals deployed in support of the military operations in Iraq and Afghanistan between September 1, 2001, and December 31, 2006.

^e ^Separation (retirement or early separation) from military service as of December 31, 2006 (excludes death as a reason for separation).

Based on all four data sources, a total of 202 deaths were identified between July 1, 2001, and December 31, 2004. Table 3 shows the proportion of total deaths contributed by each source. The VA identified the largest number of deaths, 97.0% (196/202). The SSA-DMF, separately accessed through DMDC, identified 85.6% (173/202). The NDI identified 81.2% (164/202), while the DoD-MMR accounted for less than half, 46.5% (94/202), of total deaths.

**Table 3:** Mortality Ascertainment among Millennium Cohort Study Participants by Mortality Data Source, July 1, 2001-December 31, 2004

Mortality Data Source	Deceased^a^ (*n *= 202)	Proportion of Total Deceased (%)
Department of Defense Medical Mortality Registry^b^	94	46.5
National Center for Health Statistics National Death Index^c^	164	81.2
Social Security Administration Death Master File^d^	173	85.6
Department of Veterans Affairs Mortality Data Files^e^	196	97.0

^a ^Participants who enrolled in the study between July 1, 2001, and June 30, 2003, were counted among the deceased if two of the following personal identifiers were an exact match between Millennium Cohort and mortality data sources: Social Security number, last and first name, date of birth. Deceased counts are not mutually exclusive.

^b ^Data received from the Armed Force Institute of Pathology.

^c ^Data request received by National Death Index on November 9, 2006, for death ascertainment through December 31, 2004.

^d ^Data obtained from the Defense Manpower Data Center.

^e ^Includes mortality data from the Beneficiary Identification and Records Locator Subsystem Death File, the Medical SAS Inpatient Datasets, formerly known as the Patient Treatment Files, and the Social Security Administration Death Master File.

**Figure 1:**
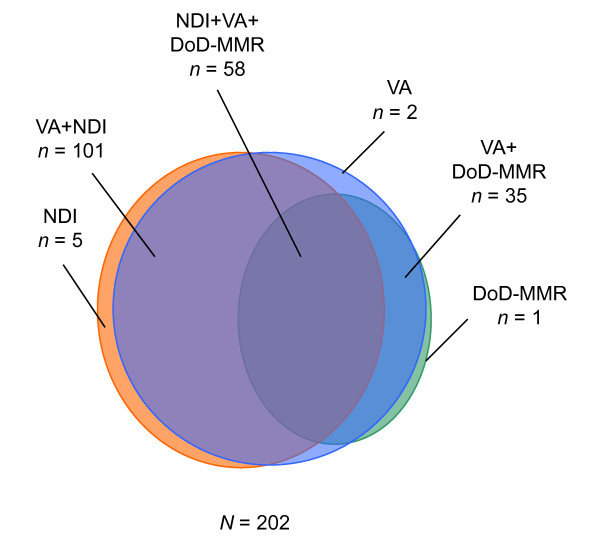
**Mortality Ascertainment based on the Department of Defense Medical Mortality Registry (DoD-MMR, n = 94), the National Death Index (NDI, n = 164), and the Department of Veterans Affairs Mortality Files (VA, n = 196), July 2001-December 2004**.

All 202 deaths were accounted for by at least one of three sources: NDI, VA files, and DoD-MMR. To illustrate the overlap between these three data sources, as well as deaths uniquely contributed by each data source, a Venn diagram was constructed (Figure 1). There was one death in the SSA-DMF (accessed through DMDC) that was not included in the VA files; however, it was recorded in NDI. The VA files missed six deaths, five identified by the NDI and one by the DoD-MMR.

The distribution of demographic and military service characteristics among decedents stratified by data source is shown in Table 4. Because the DoD-MMR is the most restrictive in terms of the source population for mortality ascertainment (active-duty military service members or Reserve/National Guard members on activated status), it is least similar to the other data sources. In contrast, the VA mortality file is most similar to the composite picture of all the data sources combined because it encompasses the SSA-DMF. The largest contrast can be seen between the DoD-MMR and NDI, with deaths identified by DoD-MMR more likely to include young, single individuals and those deployed since the onset of the conflicts in Iraq and Afghanistan and less likely to capture deaths among Reserve/National Guard members and those separated from military service.

**Table 4:** Demographic and Military Characteristics of Millennium Cohort Study Decedents by Mortality Data Source, July 1, 2001-December 31, 2004

Baseline Characteristic	All Sources (*n *= 202) %^b^	Department of Defense Medical Mortality Registry (*n *= 94) %^b^	National Center for Health Statistics National Death Index (*n *= 164) %^b^	Social Security Administration Death Master File (*n *= 173) %^b^	Department of Veterans Affairs Mortality Data Files (*n *= 196) %^b^
Gender					
Male	79.7	80.9	76.2	79.8	80.1
Female	20.3	19.2	23.8	20.2	19.9
Birth year					
Pre-1960	43.1	22.3	48.8	48.6	43.9
1960-1969	28.7	36.2	29.9	29.5	28.6
1970-1979	21.8	31.9	15.2	17.3	20.9
1980 or later	6.4	9.6	6.1	4.6	6.6
Race/ethnicity					
White non-Hispanic	74.8	69.2	74.4	75.7	75.5
Black non-Hispanic	10.4	10.6	11.0	9.3	9.7
Other	14.9	20.2	14.6	15.0	14.8
Marital status					
Not married	22.8	29.8	18.9	19.7	23.5
Married	68.8	67.0	72.0	71.1	67.9
Divorced	8.4	3.2	9.2	9.3	8.7
Education					
High school or less	50.5	48.9	50.0	48.6	51.0
Some college	24.8	22.3	26.8	25.4	24.0
Bachelor's degree	15.8	17.0	13.4	16.8	16.3
Advanced degree	8.9	11.7	9.8	9.3	8.7
Service component					
Reserve/National Guard	55.5	31.9	61.0	56.7	56.6
Active duty	44.6	68.1	39.0	43.4	43.4
Branch of service					
Army	48.5	46.8	47.6	47.4	48.5
Air Force	30.7	24.5	31.7	33.0	30.6
Navy/Coast Guard	15.8	20.2	18.3	15.6	15.8
Marine Corps	5.0	8.5	2.4	4.1	5.1
Military pay grade					
Enlisted	75.7	73.4	76.8	75.1	76.0
Officer	24.3	26.6	23.2	24.9	24.0
Occupational category					
Combat specialists	22.3	26.6	18.3	21.4	22.5
Health care specialists	9.4	8.5	11.0	11.0	9.7
Service supply and functional support	32.2	27.7	37.8	32.4	32.1
Other	36.1	37.2	32.9	35.3	35.7
Deployment experience^c^					
Pre-2001	23.3	21.3	26.2	24.9	24.0
2001-2006	15.4	25.5	7.3	13.9	15.3
Pre-2001 and 2001-2006	9.4	16.0	5.5	9.8	9.2
None	52.0	37.2	61.0	51.5	51.5
Separated from military service^d^					
No	85.6	89.4	82.9	85.6	86.2
Yes	14.4	10.6	17.1	14.5	13.8

^a ^Includes Millennium Cohort participants who enrolled in the study between July 1, 2001, and June 30, 2003, and had complete demographic data.

^b ^Percents may not sum to 100 due to rounding.

^c ^Deployment category "Pre-2001" refers to individuals deployed to the 1991 Gulf War or to Bosnia, Kosovo, or Southwest Asia between January 1, 1998, and September 30, 2000; the category "2001-2006" includes individuals deployed in support of the military operations in Iraq and Afghanistan between September 1, 2001, and December 31, 2006.

^d ^Separation (retirement or early separation) from military service as of December 31, 2006 (excludes death as a reason for separation).

## Discussion

In a recently published overview of the baseline Cohort (Panel 1) [3], proportional differences in demographic and military service characteristics between the Cohort and the probability-based original invited sample (n = 256,400) were considered small; therefore, Cohort study findings were expected to be generalizable to the military population as a whole. In this mortality study, the crude death rate for Panel 1 Cohort members was 80.7 per 100,000 person-years (95% CI: 72.2, 89.3). To put this into context, age-specific death rates for the US civilian population over the same time period (2001-2006) were all above 90 per 100,000 for age 19 years and above [27]. The lower crude death rate for our study population is consistent with a healthy worker effect since individuals entering military service must undergo extensive health screening, and accession standards ensure that only fit and healthy individuals from the general population are selected. Furthermore, periodic health examinations and fitness standards that must be met for continued active service plus universal access to medical care at military treatment facilities worldwide result in a healthy active-duty work force. When Kang and Bullman previously investigated mortality in veterans of the 1991 Gulf War era, they found that cause-specific standardized mortality ratios for both deployed and non-deployed veterans were significantly lower compared with the general US population [28].

The crude death rate for Panel 1 Cohort members is also in line with the crude death rate of 72.9 per 100,000 person-years reported for the active-duty component of the military between 1990 and 2008 by the Armed Forces Health Surveillance Center [29]. The higher crude death rate for the Millennium Cohort likely reflects that our study population includes individuals separated from military service, some potentially due to serious illnesses.

We initially compared the Panel 1 Cohort's crude death rate with that of the invited sample and found a statistically significant difference (80.7 per 100,000 person-years, 95% CI: 72.2, 89.3, vs. 101.2 per 100,000 person-years; 95% CI: 95.4, 107.0). To more fully investigate differences between the two independent groups comprising the invited sample, we compared crude death rates as well as age- and sex-adjusted overall death rates for study participants and non-participants. We also examined the distribution of mortality by demographic and military service characteristics, and finally, compared age-adjusted, category-specific death rates for these two population subgroups.

Despite higher proportions of survey respondents in older birth cohorts, the lower crude death rate for participants compared with non-participants was consistent with healthier segments of the military population having the opportunity and consenting to participate in a survey. Even more apparent was the difference between age- and sex-adjusted overall death rate for Panel 1 participants and non-participants--78.0 deaths per 100,000 person-years (95% CI: 68.3, 87.7) vs. 121.4 deaths per 100,000 person-years (95% CI: 112.9, 129.8), respectively.

In addition to healthy individuals having the opportunity to participate, more health-conscious individuals (e.g., higher educational level, married) might have greater interest in and, thus, motivation to join a health-related study. To further assess the representativeness of Panel 1 Cohort members, we compared mortality distribution by demographic and military service characteristics in Panel 1 participants and non-participants. The distribution of mortality by measured demographic factors was generally similar in the two groups. As expected, older persons and men were proportionally higher among decedents. Caucasians were overrepresented and those "not married" somewhat underrepresented among decedents in the Cohort. Among non-participants, this distribution was less pronounced but trending in the same direction. Educational level of "high school or less" was overrepresented among decedents in the non-participant group and consistent with the association between lower educational achievement and higher mortality described in the published literature [30], but this was less evident among the participants.

In addition to demographics, we examined military service characteristics to compare their distribution among Panel 1 participants and non-participants. Disproportionately higher numbers of deaths occurred in the following categories: Reserve/National Guard, Army, combat specialists, pre-2001 or no deployment experience, and not separated from active service. Army personnel and combat specialists were overrepresented among decedents, consistent with current combat operations in Iraq and Afghanistan, and may reflect the population at highest risk. Disproportionately higher numbers of deaths in Reserve/National Guard members and those with no deployment history may represent older age or certain medical conditions rendering one unfit for deployment and at the same time at higher risk of death. A similar effect, although less pronounced, was seen in the subgroup with deployment experience prior to 2001. This group included 1991 Gulf War veterans as well as veterans of conflicts in Bosnia, Kosovo, and Southwest Asia. Again, patterns of mortality by military service characteristics were generally similar when comparing Panel 1 participants and non-participants. Pay grade (officer vs. enlisted) was not an influential variable, but mortality was differentially distributed by continuing service or separation from military service. There were disproportionately fewer decedents among those separated from military service among both the participants and non-participants, which may be explained by factors such as age, service branch, and occupation. For example, a substantial proportion of young service members separate from military service after one tour of duty, and these individuals are less likely to die from disease (as opposed to external causes). Army and combat specialists were overrepresented among decedents, and since death was excluded as a reason for separation, this could contribute to disproportionately lower mortality in the separated group.

After adjustment for age, a comparison of category-specific death rates for Panel 1 participants and non-participants showed consistently higher death rates for those not participating in the study, although substantial variability in rate estimates for some categories might be the case due to small numbers. As in other health-related studies, non-participants may have less opportunity to participate due to serious illness or injury, institutionalization, or other life circumstances. Individuals who engage in unhealthy behaviors may also decide not to participate. These age-adjusted rate comparisons highlight some important differences between the two groups that warrant further investigation, notably in the categories of race/ethnicity, marital status, branch of service, and deployment experience. Next steps would include the examination of causes of death to better understand rate differences. Over time, any continued meaningful divergence in the mortality experience between the participants and non-participants should become more apparent as greater numbers accrue. Adjustment for additional covariates (survival analysis) and a focus on cause-specific rates, as well as selective subgroup analyses would be very informative.

Our final study objective was to examine the utility of selected data sources for ongoing mortality ascertainment in the Millennium Cohort. Out of the 202 total deaths identified by at least one of the four data sources, the VA databases identified the largest number (196 or 97.0%), presumably because the VA dataset also includes the SSA-DMF. In a recent study of mortality data in the VA, the BIRLS Death File alone was reported to be accurate but not complete, but with the SSA-DMF, it was viewed as the best choice if just one source had to be selected for mortality ascertainment in veteran populations [19]. The VA data files would seem to be highly suited to our study population, with all potentially eligible for death benefits from the VA and presumably all accounted for in SSA databases. If deaths were related to combat deployment, families of decedents may be more likely to claim VA death benefits, including burial at national cemeteries. Notably, the VA dataset identified two deaths not included in any of the other three data sources.

The number of deaths captured by NDI was 164 of 202 (81.2%). While we expected NDI to capture a larger proportion of the Cohort deaths, we did consider it possible that many of the deaths not captured by NDI may have occurred outside the United States and its territories (e.g., combat deaths in theaters of operations or other overseas assignments). In fact, using information from the DoD-MMR, as well as electronic military deployment data, 31 of the 38 deaths (82%) not identified by NDI were confirmed to have occurred overseas. The other seven (18%) unidentified deaths may be a result of non-matches due to inconsistencies in death certificate information and possibly longer-than-expected lag time in posting deaths. Despite missing a considerable number of Cohort deaths, NDI did identify four deaths not captured by any of the other three data sources.

The SSA-DMF was examined separately from the combined VA dataset because it is currently the main source of updates to mortality information for the Cohort (accessed through DMDC). It is also more generally accessible for mortality ascertainment than VA files. One SSA-DMF death was, in fact, not identified by the VA dataset that includes the SSA-DMF. Because mortality ascertainment is more complete in older age groups, the utility of the SSA-DMF is expected to increase over time. However, inclusion in the SSA-DMF is more likely among eligible beneficiaries either claiming death benefits or reporting a death to discontinue Social Security benefits to the deceased; thus, deaths may be incompletely captured in some population subgroups [21].

Potential sources of bias in recording or reporting of deaths by data source need to be understood and monitored over time. Additionally, changes in relative importance of currently used data sources are likely to occur as the Cohort ages and a greater proportion of individuals separate from military service, and other sources may gain importance, such as the Centers for Medicare & Medicaid Services. Finally, the success of long-term follow-up, including mortality ascertainment, is often affected by time since last follow-up and the type and amount of information available for use [10]. Thus, ongoing efforts to improve retention in the Millennium Cohort, including regular contact with participants through biennial postcards [31], provide multiple opportunities to maintain contact and correct any inconsistencies in personal identifying information.

There are several study limitations. First, due to the short period of observation (2001-2006), the total number of deaths in this relatively young, healthy occupational cohort is small (n = 341 through 2006), based on three frequently updated data sources (excluding NDI). When NDI was included, mortality could only be assessed through 2004, thus reducing the number of deaths available for the comparison of all data sources to 202. On the other hand, the large size of this Cohort with oversampling of Reserve/National Guard personnel, as well as the current involvement of US troops in combat theaters of operation, make a strong case for early assessment of mortality as an important health outcome. Over time, the number of deaths will increase, providing statistical power for cause-specific and other analyses. A second limitation is that slightly different methods were used to obtain death data because mortality ascertainment occurs on a regular basis, including prior to each survey administration, to avoid contacting family members of decedents. However, the same criteria were applied to count deaths (exact match on two of three personal identifiers). Finally, because our Cohort is composed of relatively young, healthy individuals subject to unique occupational experiences or exposures, it may be difficult to generalize our findings to other cohort studies. For example, higher likelihood of combat deaths overseas among relatively young individuals affects the degree of capture in various national mortality data sources.

Strengths of our study include using four data sources to conduct a baseline assessment of all-cause mortality in this large, population-based cohort of active and former military service members to be followed through 2022 in order to monitor any trends over time. We were able to link deployment, demographic, and separation data to the mortality data, enabling us to examine category-specific differences in the mortality experience between the participants and non-participants, as well as the four data sources. The Millennium Cohort is of particular interest to policymakers and the public because of ongoing military deployments to Iraq and Afghanistan. Many questions are being raised about potential short-term as well as long-term health effects of military service, particularly deployment. As statistical power increases, any effect of deployment or other military service-related exposures on all-cause and cause-specific mortality can be evaluated with adjustment for important covariates.

## Conclusions

In summary, early assessment of the mortality experience of the Millennium Cohort between 2001 and 2006 showed a statistically significant difference between the crude death rates of the participants and non-participants of the invited sample. Although the distribution of mortality by demographic and military service characteristics did not differ substantially between these two groups, a comparison of age-adjusted, category-specific rates highlighted some potentially important differences in early mortality experience. These findings will serve as a baseline for comparisons over time and inform future analyses focusing on cause-specific death rates to better understand mortality within our study population. Finally, available resources for mortality ascertainment were also assessed, including advantages and disadvantages. For this US military cohort, timely queries to DMDC (SSA-DMF) and AFIP (DoD-MMR) were efficient means of regularly updating vital status information at no cost. The VA was also an excellent resource, available to investigators engaging in collaborative research with VA investigators. Although restricted to the active-duty subgroup, DoD-MMR is a rich source of data on manner and circumstances of death that is not available through other sources. This additional information may be especially useful in evaluating risk factors for cause-specific mortality in order to develop prevention strategies in the active-duty component. However, NDI Plus remains the only source of information on cause-specific mortality for the entire Cohort. The advantages and disadvantages of each data source support the continued use of multiple sources for long-term follow-up, along with periodic assessments of their utility since completeness in the capture of deaths by data source is likely to change over time as Cohort members age and separate from military service.

## Abbreviations

AFIP: Armed Forces Institute of Pathology; BIRLS: Beneficiary Identification and Records Locator Subsystem; DMDC: Defense Manpower Data Center; DMF: Death Master File; DoD: Department of Defense; MMR: Medical Mortality Registry; MSID: Medical SAS Inpatient Datasets; NCHS: National Center for Health Statistics; NDI: National Death Index; SSA: Social Security Administration; VA: Department of Veterans Affairs.

## Competing interests

The authors declare that they have no competing interests.

## Authors' contributions

All authors contributed to study concept and design. TH helped direct study implementation, interpreted analytic results, and prepared major portions of the draft manuscript, as well as editing and integrating co-author comments in subsequent drafts. GG helped direct study implementation, contributed to interpretation of results, and did substantial editing for content. CL was the major contributor to study implementation, performed all analyses, drafted a portion of methods, and edited the manuscript for content. EB drafted a section of the discussion and edited the manuscript for content. LP drafted a section of methods and edited for content. BS assisted with study implementation and edited the manuscript for content. PA helped interpret results and edited the manuscript for content. Finally, TS helped oversee analytic activities and edited the manuscript for content. All authors read and approved the final manuscript.
